# Evaluation of Caries Risk Using Cariogram Among Orthodontic Patients Before and During Treatment: A Comparative Study

**DOI:** 10.7759/cureus.63745

**Published:** 2024-07-03

**Authors:** Navin Oommen Thomas, Veena Nagappan Kamalabhai, Sona Joseph, Rini Rajendran, Moushmi Chalakkarayil Bhagavaldas, Elashri Chatterjee

**Affiliations:** 1 Department of Orthodontics and Dentofacial Orthopedics, Pushpagiri College of Dental Sciences, Thiruvalla, IND; 2 Department of Orthodontics and Dentofacial Orthopedics, Sri Sankara Dental College, Thiruvananthapuram, IND; 3 Department of Conservative Dentistry and Endodontics, Mahe Institute of Dental Sciences and Hospital, Mahe, IND; 4 Department of Conservative Dentistry and Endodontics, Azeezia College of Dental Sciences and Research, Kollam, IND; 5 Department of Conservative Dentistry and Endodontics, Al-Azhar Dental College, Thodupuzha, IND; 6 Department of Periodontology, Hitkarini Dental College and Hospital, Jabalpur, IND

**Keywords:** streptococcus mutans, orthodontic treatment, fluoridated toothpaste, dental caries, cariogram

## Abstract

Objective

The primary objective was to examine the Cariogram parameters among orthodontic patients with fixed appliances and evaluate the impact of preventive measures on mitigating the risk of dental caries during orthodontic therapy.

Materials and methods

Patients visiting the representative orthodontic clinics across 14 districts of Kerala participated in the comparative cross-sectional study from January 2023 to January 2024. The sampling method employed in this study was convenience quota sampling, where study subjects were allocated from each of the low, moderate, and high caries risk profiles until the sample size reached the minimal requirement within each group. The baseline Cariogram scores were used to divide the consented participants into two distinct groups. The intervention group was provided with preventive initiatives, including toothpaste comprising 1,450 ppm fluoride, 0.2% NaF mouthwash, pre-cut strands of SuperFloss, an orthodontic toothbrush designed for orthodontic braces, and an interdental flexible brush, as well as videos, pamphlets, and brochures that promoted oral health habits. In contrast, the control group received normal oral health education solely through the use of pamphlets and brochures. After six months, the Cariogram elements were re-evaluated for individuals in both groups. The independent sample t-test and paired t-test were applied to evaluate statistically significant differences between and within the two groups, respectively, using IBM SPSS Statistics for Windows, Version 26 (Released 2019; IBM Corp., Armonk, NY, US). The distribution of patients based on their caries risk profiles was compared between groups at the commencement of the study and six months later using the Chi-square test.

Results

While the intervention group had 20 males and 22 females, the control group consisted of 21 males and 21 females. The average age in the intervention and control groups was 20.7±3.56 years and 21.2±3.12 years, respectively. Between the two groups, age (t=-0.68; p=0.50) and gender differences (λ^2^=0.05; p=0.83) were statistically insignificant. The percentage mean of the "Chance to avoid caries" associated with the intervention group increased significantly from 46.15±0.96 to 57.88±1.91, (p<0.001). On the other hand, the chance to avoid caries in the control group at the commencement of the treatment and six months later was found to be statistically insignificant. A statistically highly significant differences for all the Cariogram parameters were found when contrasted between the groups after six months of orthodontic treatment. The distribution of caries risk categorization between the control and intervention groups after six months of orthodontic treatment was found to be statistically significant (λ^2^=20.16; p<0.0001). Further, a statistically significant difference was observed during the pre-treatment phase and six months later in the intervention group (λ^2^=13.02; p=0.001).

Conclusion

The study findings reveal that it would be prudent to utilize 0.2% sodium fluoride mouth rinse, SuperFloss, an orthodontic toothbrush designed for orthodontic braces, and an interdental flexible brush, along with toothpaste containing 1450 ppm fluoride daily, to mitigate the risk of dental cavities during orthodontic treatment, in comparison to the control group.

## Introduction

The goal of orthodontic therapy is to establish a long-lasting, harmonious occlusion by correcting the bite with orthodontic appliances [1]. Fixed appliances foster a greater cariogenic burden surrounding orthodontic brackets and bands because they not only enhance the rate of biofilm production but also the number of acid-producing microbes within the biofilm. White spot lesions (WSLs) and demineralization of enamel will eventually occur from the acidogenic bacteria in the dental biofilm if patients are unable to maintain proper oral hygiene during orthodontic therapy [2]. Fixed appliance therapy has become an essential component of contemporary orthodontics, but it has also been linked with specific negative consequences such as periodontal damage, root resorption, caries, etc. The local proliferation of *Streptococcus mutans* (*S. mutans*) is favoured by the formation of plaque-retentive regions in the vicinity of fixed orthodontic appliances, leading to an upsurge in cariogenic organisms in the saliva [3].

WSLs are particularly notable because of their detrimental effect on visual appearance and their potential to develop into carious lesions [4]. The decrease in dental cavities in the majority of developed nations during the preceding 40 years has mostly been ascribed to the regular usage of fluoride dentifrices. This can be primarily attributed to the involvement of fluoride in the processes of demineralization and remineralization, as well as the antibacterial properties exhibited by the fluoride component [5]. Caries risk assessment (CRA) programmes are universally advocated in contemporary dentistry to manage caries. There are currently multiple CRA methods that are based on reasoning and algorithms, and they have different numbers of quantitative caries risk variables. Questionnaires and clinical investigations, such as salivary and microbiological assessments, can be used to evaluate the risk variables. CRA programmes encompass several characteristics associated with caries and assist clinicians in evaluating a patient's risk profile and making informed decisions regarding prevention, treatment, and follow-up intervals [6,7].

Despite the existence of multiple factors that have shown significant connections with prospective caries, no solitary test can reliably anticipate an individual's vulnerability to caries [3]. Nevertheless, upon analyzing several caries-associated characteristics using a computer-based software known as the Cariogram [8], an apparent correlation appears to exist between the depicted caries risk over the years for both children and adults [9,10], and among those undergoing orthodontic treatment [11]. Cariogram is a computer-based CRA model that calculates aggregated caries risk for future caries utilizing a weighted study of many etiological characteristics of the individual in question [8,12]. It is an interactive application that provides a visual representation of the CRA. Hence, the primary objective was to examine the Cariogram parameters among orthodontic patients with fixed appliances and evaluate the impact of preventive measures on mitigating the risk of dental caries during orthodontic therapy.

## Materials and methods

Study design and study population

Patients visiting the representative orthodontic clinics across 14 districts of Kerala participated in the comparative cross-sectional study over one year. The study was approved by the Institutional Ethics Committee of the Pushpagiri College of Dental Sciences, Thiruvalla, India (PCDS/IEC/J 12/11/16). Before commencing the examination, informed consent was sought. The sampling method employed in this study was convenience quota sampling, whereby the study subjects were allocated from each of the low, moderate, and high caries risk profiles till the required sample size was met within each group.

A preliminary study was carried out on 10 participants from each category (Group 1: those receiving orthodontic therapy, and Group 2: those about to begin treatment) to assess the practicality of the study, the duration needed for examining each participant, to get acquainted with the laboratory protocol for salivary analysis, and to calculate the sample size.

The sample size (n) was determined from the pilot study findings, taking into account a 95% confidence interval, a 5% significance level, and an 80% power of the study. This determination was made using the following formula:

n = (S_1_^2^+S_2_^2^)(Z_1-α/2_+Z_1-β_)^2^/(X_1_-X_2_)^2^,

where S_1_ is the standard deviation of Group 1 (3.93), S_2_ is the standard deviation of Group 2 (3.76), and X_1_-X_2_ is the mean difference between the chance to avoid caries between the two groups (3.03) as estimated using the Cariogram software v3.0 (Malmö University, Malmö, Sweden). The minimum estimated sample size was 77. However, the sample size was inflated to 84 (42 in each group) to eliminate the attrition bias. Six subjects (three in each group) were chosen from each of the 14 districts in Kerala. The representative orthodontic clinics were selected randomly from the list of clinics from 14 districts using a simple random sampling method.

The inclusion criteria encompassed individuals classified as orthodontic patients who were 18 years of age or older, able to communicate and comprehend their local language, and required fixed orthodontic therapy in maxillary and mandibular arches for a minimum duration of six months. The exclusion criteria encompassed individuals with periodontal conditions, cleft lip and palate or other syndromic conditions, systemic disorders, as well as people who smoke or take drugs that may alter the natural oral flora or salivary flow such as atropine, scopolamine, etc.

In its entirety, the current investigation comprised three distinct phases: (A) The Cariogram tool was utilized to establish the caries risk profile for each patient. The patients were grouped into three categories: low risk (61 to 80%), medium risk (41 to 60%), and high risk (0 to 40%). The caries risk profile for every individual was determined by considering the extent of the sector - the chance to avoid caries. (B) The baseline Cariogram scores were used to divide the consented participants into two distinct groups. Each group comprised people with low, moderate, and high levels of risk, as identified in the preceding step. The intervention group was provided with preventive initiatives, including toothpaste comprising 1,450 ppm fluoride, 0.2% NaF mouthwash, pre-cut strands of SuperFloss, an orthodontic toothbrush designed for orthodontic braces, and an interdental flexible brush, as well as videos, pamphlets, and brochures that promoted oral health habits. In contrast, the control group received normal oral health education solely through the use of pamphlets and brochures. (C) After six months, the Cariogram elements were re-evaluated for individuals in both groups.

All subjects completed the usual Cariogram questionnaire. The manual (Table 1) was used to rank all nine caries-associated characteristics on a scale of 0 to 2 or 0 to 3. Subsequently, all the data were entered into the Cariogram software for visual illustration that accurately depicts the probability of preventing new caries lesions as percentages. All patients were assigned a score of 1 to the 10th element, known as "clinical judgment," indicating that the caries risk was assessed based on other Cariogram parameters.

**Table 1:** Caries-associated Cariogram parameters DMFT: decayed, missing, and filled teeth

Sector	Factor	Data collected	Cariogram scores
Circumstances	Caries experience	Previous caries experience, such as cavities, fillings, and tooth loss caused by caries. Non-cavitated white spot lesions were evaluated according to modified WHO dentition status through dental exams and bitewing radiography.	0: DMFT 0
			1: DMFT 1
			2: DMFT 2
			3: DMFT 3 or more
Diet	Diet and its contents	Lactobacillus count was utilized to assess the cariogenicity of meals, including the sugar level.	0: <103 CFU/mL
			1: 104 CFU/mL
			2: 105 CFU/mL
	Diet frequency	The estimated amount average number of snacks and meals per day (average of three days' food diary)	0: 3 meals/day
			1: 4-5 meals/day
			2: 6-7 meals/day
			3: >7 meals/day
Bacteria	Plaque amount	Assessment of oral hygiene using the Silness-Löe plaque index	0: 0 (Excellent)
			1: 0.1 to 0.9 (Good)
			2: 1.0 to 1.9 (Fair)
			3: 2.0 to 3.0 (Poor)
	Streptococcus mutans	Salivary pathogens were quantified as Colony Forming Units (CFU) per millilitre of saliva.	0: 0-103 CFU/mL
			1: 103-104 CFU/mL
			2: 104-105 CFU/mL
			3: >105 CFU/mL
Susceptibility	Fluoride programme	Estimating the amount of fluoride available in the oral cavity over a period using the interview method.	0: Maximum fluoride programme
			1: Fluoride supplements
			2: Only fluoride toothpaste
			3: No fluoride
	Salivary secretion	Estimate the quantity of stimulated salivary production presented as mL of saliva per minute.	0: >1.1 mL/min
			1: 0.9-1.1 mL/min
			2: 0.5-0.9 mL/min
			3: <0.5 mL/min
	Salivary buffering capacity	Estimation of buffering capacity to buffer acids	0: pH≥6.0
			1: pH 4.5-5.5
			2: pH≤4.0

Cariogram tool parameters

Caries Experience

A dental chair was utilized to perform the clinical evaluation, employing a mouth mirror, standard illumination, and a WHO probe. The scoring of caries was conducted following the criteria established by the WHO, employing the DMFT index, which measures the number of decayed, missing, and filled teeth. Furthermore, a solitary researcher who had received proper training and calibration conducted all oral examinations. The teeth were dried using compressed air. A comprehensive evaluation and diagnosis of initial caries lesions were performed by combining visual inspection, tactile probing, clinical photographs, and radiographic analysis (the radiographic evaluation was undertaken whenever required). Consequently, the oral examination of a sample of 10 subjects was performed on two different occasions to assess the intraexaminer reliability using Kappa statistics, and the Kappa coefficient of 0.86 indicated a high level of reliability.

Estimation of Salivary Parameters

Clear instructions to abstain from eating and using mouthwash before one hour were given to every individual having their saliva sampled. They were instructed to meticulously rinse their mouth with water to prevent contamination from food debris. The salivary flow rate was measured by obtaining a stimulated saliva specimen by chewing an unflavoured piece of paraffin wax for five minutes during the morning hours, specifically between 10:00 AM and 11:30 AM, consequently order to maintain the circadian rhythm. The study participants were instructed to expel saliva into a funnel that was linked to a calibrated test tube at one-minute intervals for five minutes. The salivary volume was then measured in millilitres per minute. The salivary buffering capacity was estimated using the colorimetric method with Indikrom papers (Table 1).

Estimation of Microbiological Parameters

The specimen was streaked on mitis salivarius-bacitracin agar medium (HiMedia Laboratory, Mumbai, India), which is known for its selectivity towards mutans *Streptococci*, and on Rogosa SL agar medium (HiMedia Laboratory, Mumbai, India), which is specifically designed for *Lactobacilli*, employing a loop to inoculate. The counts of colonies were conducted through an electron microscope (Leo 1530 Gemini, Oberkochen, Germany) and quantified in terms of the number of Colony Forming Units (CFU) per millilitre of saliva and a score of 0 to 3 was assigned.

Cariogram assessment

A single researcher conducted examinations on all patients. The data collection process followed the Cariogram tool, which involved gathering information on many factors such as prior health history, dietary habits, plaque index, colony-forming units of *S. mutans* and *Lactobacillus*, fluoride supplementation, and salivary specimens for assessing flow rate and buffering potential. Following the bonding of the brackets, the primary investigator provided clinical standards and routine oral hygiene instructions to both the intervention and control groups of participants. In addition, they were provided with an additional pamphlet on health promotion. The preventative programmes were introduced to the patients in the intervention category during the initial session, following the bonding of brackets. The investigator provided these programmes as outlined as follows.

The significance of regular dental care lies in its ability to detect white spots, which indicate incipient caries in individuals. The provision of nutritional guidance includes dietary modifications, such as the reduction of meal and snack frequency, decreased carbohydrate intake, and increased consumption of foods high in fibre. The study included five products: maximum cavity protection toothpaste with a fluoride concentration of 1,450 ppm, multi-protection mouthwash (Oral-B Pro-Expert Multi-Protection Mouthwash; Procter & Gamble, Schwalbach am Taunus, Germany), pre-cut strands of SuperFloss, an orthodontic toothbrush designed for orthodontic braces, and an interdental flexible brush. The multi-protection mouthwash used in the study preserves good oral health and lessens plaque by eliminating bacteria that cause plaque formation. It contains sodium fluoride (an anti-cariogenic agent), methylparaben (prevents microbial growth), cetylpyridinium chloride (boosts oral hygiene by attenuating gingival inflammation, mitigating plaque maturation, and preventing bacterial growth), glycerine, and other preservatives and sweetening agents.

It is advisable to promote consistent oral hygiene by utilizing fluoridated toothpaste, twice a day, ideally in the morning before breakfast and at night before going to bed. Additionally, it is recommended to employ 0.12% chlorhexidine mouthwash twice daily, with each application involving a 30-second gargling motion of the mouth. The investigator also presented videos and images that focused on the correct brushing technique, interdental toothbrushes, and the use of SuperFloss. The categories of risk (low/moderate/high) among those undergoing fixed orthodontic therapy were determined using the Cariogram software. This software utilizes algorithms to assess the provided data and portrays its findings in a pie diagram.

The chart illustrates five distinct groups of factors associated with dental caries, represented as percentages: (1) The variable "Diet" was determined by considering the combined effects of the amount of sugar consumed and the number of *S. mutans* and *Lactobacilli*, represented by the dark blue sector. (2) The variable "Bacteria" was determined by combining the plaque score and the number of *S. mutans*. (3) The "Susceptibility" includes factors such as the fluoride programme, rate of salivary secretion, and buffering capacity. (4) The "Circumstances" were determined based on previous caries experiences. (5) The "Chance to avoid caries" sector represents the likelihood of preventing caries.

The investigator examined the patients belonging to the intervention category across six months of orthodontic therapy, where they were requested to exhibit appropriate use of toothbrushes and dental floss. The evaluation of the study participants in both groups, six months after the initiation of orthodontic therapy, was executed, and a Cariogram plot was generated to assess the impact of the procedure.

Statistical analysis

IBM SPSS Statistics for Windows, Version 26 (Released 2019; IBM Corp., Armonk, NY, US), was utilized to analyze the data. Descriptive statistics were computed in both groups for all factors. The independent sample t-test was employed to ascertain statistically significant differences between the two groups. Additionally, a paired t-test was employed to identify statistically significant differences within the group before orthodontic therapy and six months following the course of therapy. The distribution of patients based on their caries risk profiles was compared between groups at the commencement of the study and six months later using the Chi-square test.

## Results

**Figure 1:**
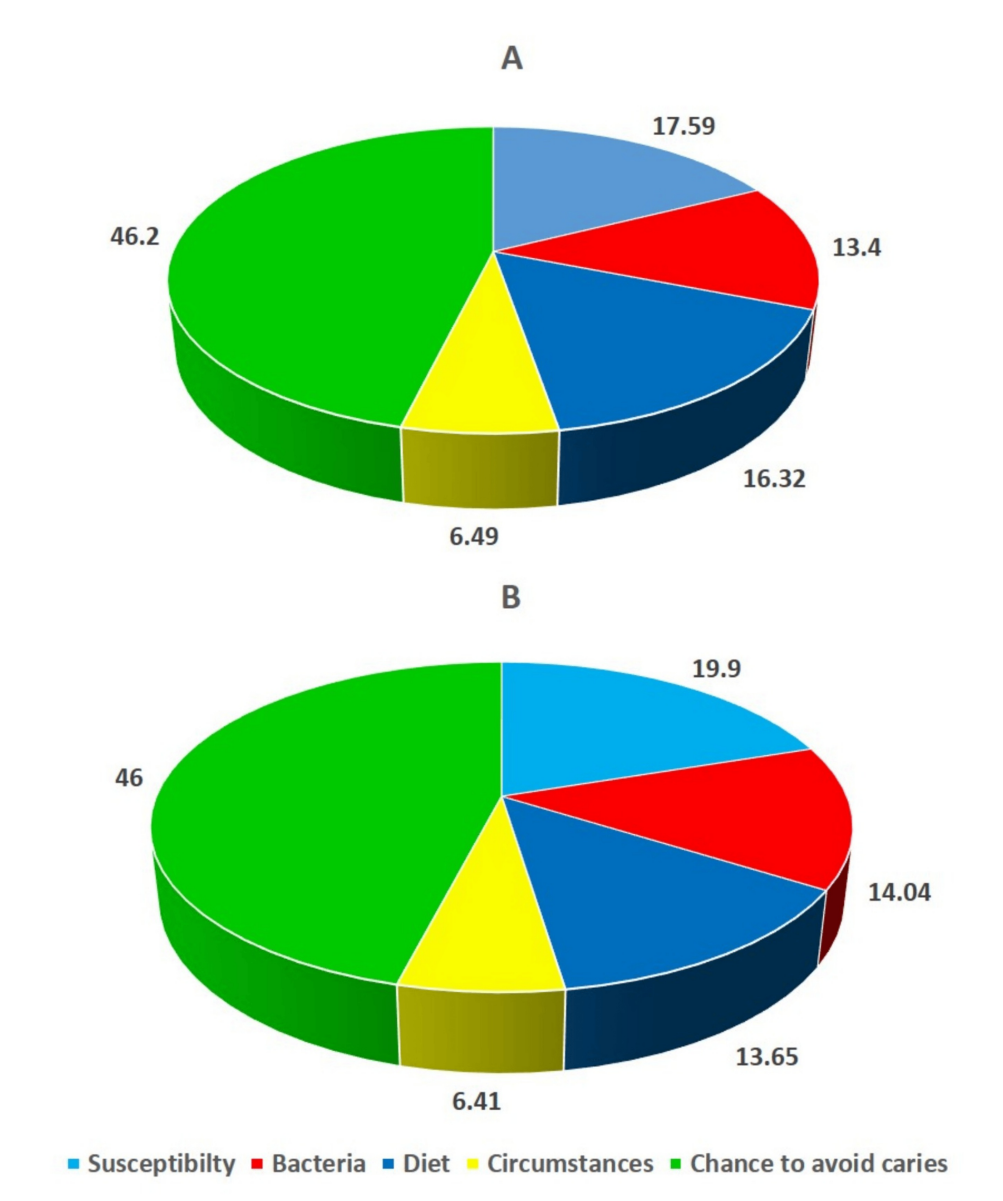
Cariogram models of the control group at the pre-treatment phase and six months following orthodontic treatment A) Pre-treatment cariogram; B) Cariogram after six months

**Figure 2:**
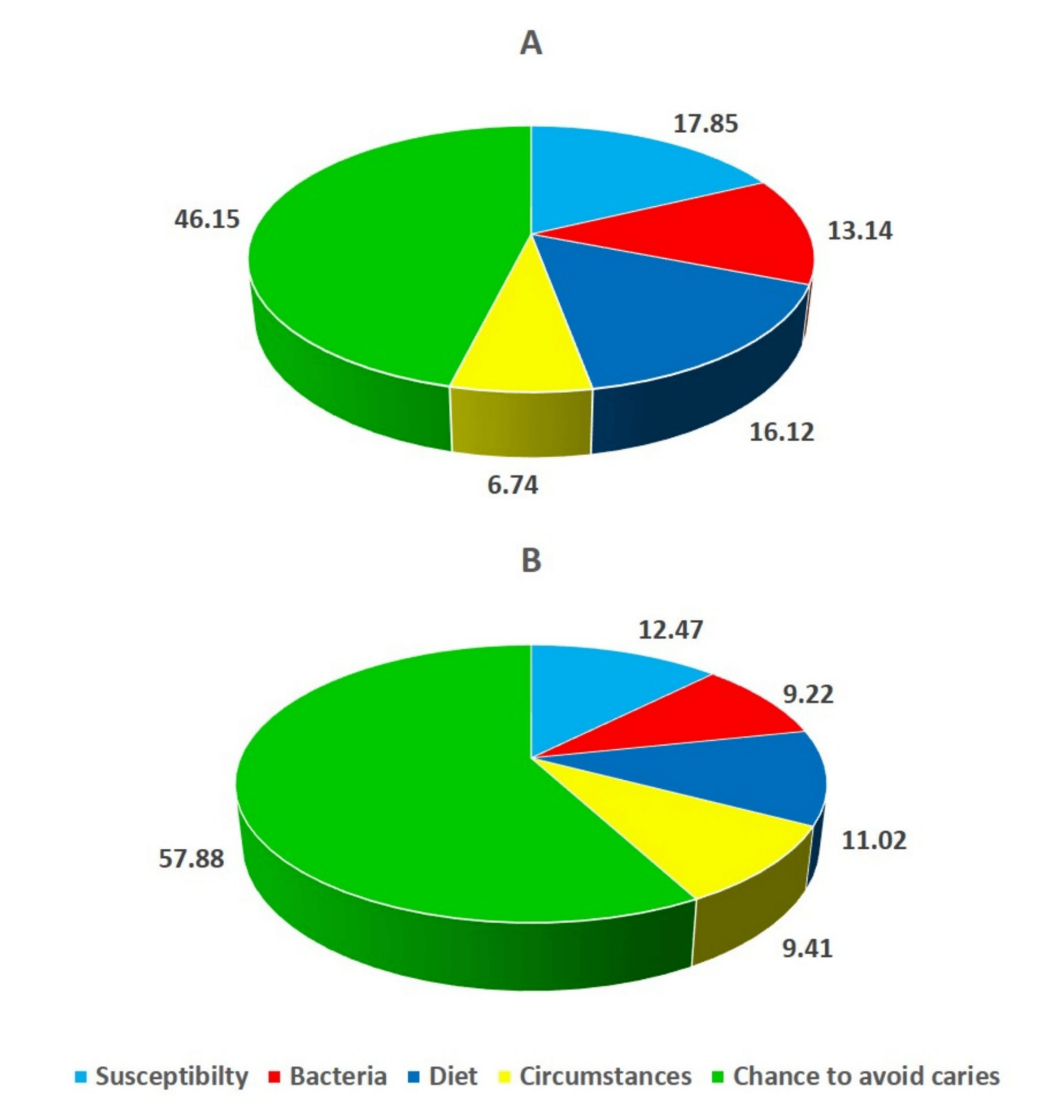
Cariogram model of the intervention group at the pre-treatment phase and six months following orthodontic treatment A) Pre-treatment cariogram; B) Cariogram after six months

While the intervention group had 20 males and 22 females, the control group consisted of 21 males and 21 females. The age of the subjects ranged from 19 to 28 years old, with average age in the intervention and control groups were 20.7±3.56 years and 21.2±3.12 years, respectively. Between the two groups, age (t=-0.68; p=0.50) and gender differences (λ^2^=0.05; p=0.83) were statistically insignificant using unpaired t-test and Chi-square test, respectively. Following six months during the orthodontic treatment, two subjects in the control group and one in the intervention group were lost to follow-up. The Cariogram profile and associated parameters are presented in Figures 1-2 for the control and intervention groups during the pre-treatment period and six months after the orthodontic therapy.

Table 2 represents the Cariogram caries-associated parameters that were contrasted between the two groups at the start of the therapy and six months later. The percentage mean of the "Actual chance to avoid caries" associated with the intervention group increased significantly from 46.15±0.96 to 57.88±1.91, with a statistically significant difference (p<0.001). Other characteristics like "Diet," "Bacteria," and "Susceptibility" had average values of 16.12±0.93, 13.14±1.25, and 17.85±0.7; these values subsequently dropped to 11.02±2.17, 9.22±2.18, and 12.47±1.48, respectively (p<0.0001). However, the "Circumstances" sector showed an upsurge in the average value from 6.74±0.96 to 9.41±0.94 in the intervention group. On the other hand, the chance to avoid caries in the control group at the commencement of the treatment and six months later was found to be statistically insignificant. The study employed the independent sample t-test to examine any significant variations in the Cariogram parameter scores between the two groups at baseline and after a six-month follow-up. A statistically significant difference for all the Cariogram parameters was found when contrasted between the groups after six months of orthodontic treatment.

**Table 2:** Comparative evaluation between the two groups based on Cariogram parameters at the commencement of the orthodontic treatment and after six months * Indicates a significant difference at p<0.05 The independent sample t-test and paired t-test are applied to evaluate statistically significant differences between and within the two groups, respectively

Variable	Interval	Control group (n=40)	Intervention group (n=41)	Intergroup p-value
Chances to avoid caries	T0	46.2±1.19	46.15±0.96	0.84
	T1	46±1.46	57.88±1.91	<0.001*
Intragroup p-value		0.53	<0.001*	
Diet	T0	16.32±1.13	16.12±0.93	0.39
	T1	13.65±1	11.02±2.17	<0.001*
Intragroup p-value		<0.001*	<0.001*	
Bacteria	T0	13.4±0.64	13.14±1.25	0.24
	T1	14.04±1.11	9.22±2.18	<0.001*
Intragroup p-value		0.003*	<0.001*	
Susceptibility	T0	17.59±0.75	17.85±0.7	0.11
	T1	19.9±0.87	12.47±1.48	<0.001*
Intragroup p-value		<0.001*	<0.001*	
Circumstances	T0	6.49±1.18	6.74±0.96	0.3
	T1	6.41±1.01	9.41±0.94	<0.001*
Intragroup p-value		0.71	<0.001*	

**Figure 3:**
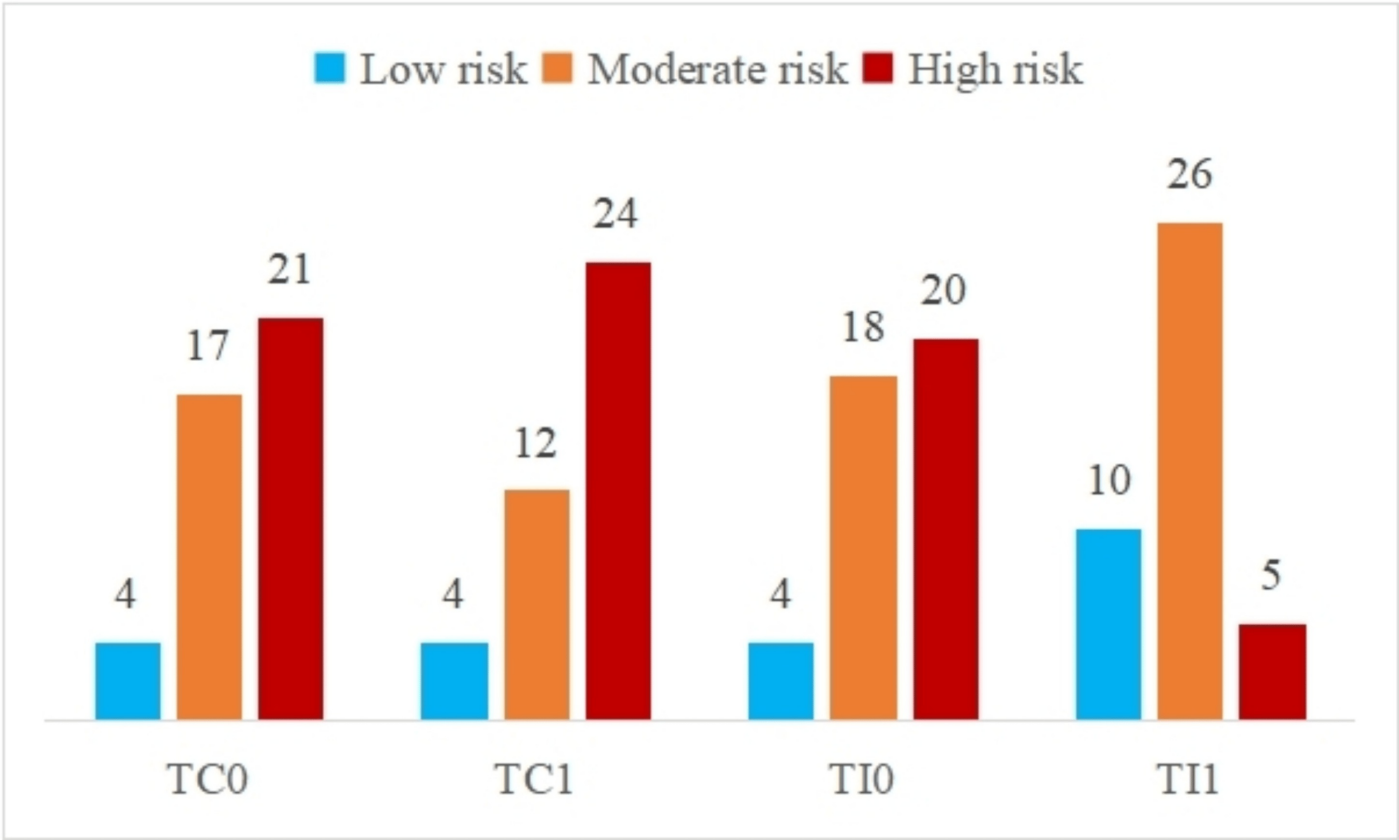
Distribution of caries risk categories based on Cariogram parameters during the pre-treatment phase and six months after orthodontic treatment in the control and intervention groups TC0: control group at pre-treatment phase; TC1: control group during orthodontic treatment; TI0: intervention group at pre-treatment phase; TI1: intervention group during orthodontic treatment; TC0 vs TI0: λ^2^=0.05, p=0.97; TC0 vs TC1: λ^2^=1.01, p=0.6; TI0 vs TI1: λ^2^=13.02, p=0.001; TC1 vs TI1: λ^2^=20.17, p<0.001

Figure 3 shows the distribution of caries risk categories based on Cariogram parameters during the pre-treatment phase and six months after orthodontic treatment in the control and intervention groups.

Additionally, in the pre-treatment period, the intervention group consisted of four subjects (9.52%) at low risk, 18 with moderate risk (42.86%), and 20 subjects (47.62%) with high risk. Conversely, the control group during the pre-treatment phase, comprised four patients who were classified as low risk (9.52%), 17 at moderate risk (40.48%), and 21 at high risk (50%). In the intervention group, following six months, the Cariogram tool showed that 12.2% (n=5) had a high caries risk, 63.41% (n=26) had a moderate caries risk, and 24.39% (n=10) had a low caries risk. As shown in Figure 3, the risk categorization distribution in the control group after six months of treatment was as follows: 60% (n=24) had a high caries risk, 30% (n=12) had a moderate risk, and 10% (n=4) had a low caries risk. The difference in the distribution of caries risk categorization between the control and intervention groups after six months of orthodontic treatment was found to be statistically significant (λ^2^=20.16; p<0.0001). Further, a statistically significant difference was found during the pre-treatment phase and six months later in the intervention group (λ^2^=13.02; p=0.001). 

## Discussion

The current study emphasizes evaluating caries risk in individuals receiving orthodontic therapy and suggests prophylactic interventions to lower the incidence of new dental caries or WSLs. The findings revealed a significant difference in the percentages of chances to avoid caries in the Cariogram between the two groups. Additionally, the study indicated that consistent and accurate utilization of toothpaste with a fluoride concentration of 1,450 ppm, mouthwash, SuperFloss, interdental toothbrush, and orthodontic floss, when combined with appropriate health education, can effectively mitigate the likelihood of caries, particularly among orthodontic patients who are highly vulnerable owing to the likelihood of accumulation of plaque.

According to Karabekiroglu and Ünlü [13], a duration of 12 weeks is deemed sufficient for the detection of the benefits of preventive procedures. However, alternative research suggests that a minimum duration of six months is preferred for the identification of the outcomes of caries preventive measures. Therefore, the current investigation was carried out for six months, aligning with the investigation completed by Doost-Hoseini et al. [2]. This study confirms that the usage of 1,450 ppm fluoridated toothpaste leads to a lowering of the high caries risk profile, as indicated by the Cariogram pie chart. This finding aligns with the results reported by Karabekiroglu and Ünlü [13], who utilized a Cariogram to assess the efficacy of 1,450 ppm fluoridated toothpaste, fluoride varnish, and chlorhexidine over 12 weeks among adolescents. Furthermore, the outcomes of the current investigation align with the conclusions of Al Mulla et al. [11], who examined caries-associated variables in individuals receiving fixed orthodontic therapy for six months, employing a Cariogram as a diagnostic tool, despite the absence of any therapies. Additionally, a different study documented that the Cariogram model could be employed for those undergoing orthodontic treatment with or without salivary examinations [12].

Despite a decrease in dietary frequency, the control group's caries risk was similar before orthodontic therapy and six months following the treatment owing to a marked rise in the quantity of cariogenic microorganisms and the absence of additional fluoride prophylaxis. This finding is congruent with previous research that has shown a correlation between the use of fixed appliances and an elevated level of cariogenic microbes [1,14]. Additionally, prior research has demonstrated a positive association between acidogenic bacterial species, including *S. mutans* and *Lactobacilli*, and the occurrence of dental caries [15,16]. Hence, it is advantageous to reduce the concentrations of these cariogenic pathogens during orthodontic therapy. Patients who fail to prioritize their well-being and oral hygiene during their treatment may experience alterations in the colour surrounding brackets, eventually resulting in the development of caries during their treatment [17]. Hence, the timely identification and evaluation of caries, encompassing the analysis of salivary bacterial counts, might assist orthodontists in providing tailored suggestions to mitigate the likelihood of dental caries [3]. In contemporary caries care, patients are managed based on their risk level, which can be categorized as low, moderate, or high. Additionally, initial lesions on tooth surfaces are to be meticulously tracked [2]. In addition, Mannaa et al. [5] determined that using toothpaste containing 5,000 ppm fluoride for six weeks can effectively decrease the risk of caries. This can be proved by the Cariogram tool, as it increases the likelihood of preventing caries. 

Fixed braces have the potential to trap food-related debris, hence posing challenges to oral hygiene practices. In the vicinity of the gingival margin and close contact with orthodontic bands and brackets, there is an increased tendency for plaque to adhere [18]. A randomized clinical trial revealed that the test group, which used toothpaste alongside fluoride varnish, had significantly decreased visible plaque index scores following de-bonding, in comparison to the control group, which only employed toothpaste [19]. The optimal dental health of the individuals is an essential requirement for orthodontic treatment acceptance, which includes reduced caries activity and adequate oral hygiene. Additionally, it is established that fluoride supplements did not have any impact on plaque levels [14].

The study findings demonstrate the efficacy of caries risk evaluations and personalized caries prevention measures as an appropriate strategy for managing dental caries. To validate the efficacy of preventive measures for patients receiving fixed orthodontic therapy, future investigation, and randomized controlled trials are warranted for a comparative analysis of extensive samples obtained from diverse governmental and private orthodontic centres, encompassing individuals from varying socio-economic backgrounds.

Limitations

The study's focus on assessing caries risk before and during orthodontic treatment might overlook other factors influencing oral health during this period. While Cariogram is a useful tool for assessing caries risk, it has its limitations. The predictive accuracy of a Cariogram may vary based on factors such as the population being studied, the availability of accurate data inputs, and the tool's calibration to specific patient populations.

## Conclusions

The intervention effectively increased the chance of avoiding caries and significantly reduced key risk factors such as diet, bacteria, and susceptibility in the intervention group. The improvement in caries risk categorization further underscores the intervention's success. The increase in the "Circumstances" parameter suggests the need for further exploration of external factors that may influence caries risk. These results highlight the importance and effectiveness of targeted interventions in managing caries risk during orthodontic treatment. Future research could focus on refining these interventions and addressing external factors to enhance caries prevention strategies further.
